# After “The China Virus” Went Viral: Racially Charged Coronavirus Coverage and Trends in Bias Against Asian Americans

**DOI:** 10.1177/1090198120957949

**Published:** 2020-09-10

**Authors:** Sean Darling-Hammond, Eli K. Michaels, Amani M. Allen, David H. Chae, Marilyn D. Thomas, Thu T. Nguyen, Mahasin M. Mujahid, Rucker C. Johnson

**Affiliations:** 1 University of California, Berkeley, CA, USA; 2 Tulane School of Public Health and Tropical Medicine, New Orleans, LA, USA; 3 University of California, San Francisco, CA, USA

**Keywords:** COVID-19, coronavirus, media effects, implicit bias, racism, Asian American

## Abstract

On March 8, 2020, there was a 650% increase in Twitter retweets using the term “Chinese virus” and related terms. On March 9, there was an 800% increase in the use of these terms in conservative news media articles. Using data from non-Asian respondents of the Project Implicit “Asian Implicit Association Test” from 2007–2020 (*n* = 339,063), we sought to ascertain if this change in media tone increased bias against Asian Americans. Local polynomial regression and interrupted time-series analyses revealed that Implicit Americanness Bias—or the subconscious belief that European American individuals are more “American” than Asian American individuals—declined steadily from 2007 through early 2020 but reversed trend and began to increase on March 8, following the increase in stigmatizing language in conservative media outlets. The trend reversal in bias was more pronounced among conservative individuals. This research provides evidence that the use of stigmatizing language increased subconscious beliefs that Asian Americans are “perpetual foreigners.” Given research that perpetual foreigner bias can beget discriminatory behavior and that experiencing discrimination is associated with adverse mental and physical health outcomes, this research sounds an alarm about the effects of stigmatizing media on the health and welfare of Asian Americans.

## Coronavirus Media Coverage

On February 11, 2020, the World Health Organization (WHO) advised that media institutions refer to the novel coronavirus as “COVID-19” or coronavirus. “Don’t” they continued, “attach locations or ethnicity to the disease, this is not a ‘Wuhan Virus,’ ‘Chinese Virus’ or ‘Asian Virus.’ The official name for the disease was deliberately chosen to avoid stigmatisation” (WHO, 2020, p. 2). As the Atlantic Council’s Digital Forensic Lab (DFR) illustrates in a detailed timeline of public discourse relating to the coronavirus (Table 1; Rizzuto, 2020), most media outlets quickly complied with these guidelines. But in March, prominent Republican elected officials (initially Mike Pompeo and Paul Gosar) and conservative media outlets began using stigmatizing language by associating the virus with China. As DFR explains, on March 8 there was a 650% increase in Twitter retweets using the term “Chinese virus” and related terms. On March 9, there was an 800% increase in the use of these terms in news media articles. The terms were repeated by myriad conservative social and news media channels throughout the month thereafter (Rizzuto, 2020).

**Table 1.** 2020 Time Line of Tone of COVID-19 Coverage.

Date	Media coverage
January 30	WHO declares coronavirus an international emergency.
February 11	WHO provides guidance to use the terms “coronavirus” and “COVID-19,” and to avoid “stigmatizing” terminology.
February 12–March 6	The use of stigmatizing terminology falls to negligible levels in Twitter retweets and news media articles.
March 7	Mike Pompeo uses “Chinese virus” terminology on *Fox and Friends* and CNBC.
March 8	Republican Paul Gosar tweets about “Wuhan virus.”
March 8	There is a 650% increase (compared to highest reported prior daily average) in Twitter retweets with terms “Chinese virus,” “Wuhan virus,” “Chinese coronavirus,” and “Wuhan coronavirus.
March 9	There is an 800% increase (compared to prior day) in number of online news articles using stigmatizing terminology.

*Note.* WHO = World Health Organization.

The temporal discontinuity in the nature of media regarding coronavirus can be leveraged to study whether the media tone shift resulted in an increase in bias against Asian Americans. This is an urgent question. A newly created database shows that Asian Americans have suffered 2,583 COVID-related hate crimes and acts of discrimination since March 19, 2020 (Chinese for Affirmative Action and Asian Pacific Policy & Planning Council, 2020), and research has linked experiences of discrimination with multiple adverse mental and physical health outcomes among Asian Americans (Gee et al., 2007; Gee et al., 2009).

## Media Effects on Racial Bias

Theories of “media effects” suggest that the use of stigmatizing terms such as the “Chinese virus” could negatively influence public attitudes about Asian Americans. Media effects are defined as “changes in cognitions, emotions, attitudes, and behavior that result from media use” (Valkenburg et al., 2016, p. 338). Research indicates that consuming media that depicts stigmatized groups in a stereotypical or threatening manner can increase racial bias (Arendt, 2013; Dasgupta, 2013; Tukachinsky et al., 2015; Valkenburg et al., 2016; Wetts & Willer, 2019; Yogeeswaran et al., 2012). One pernicious stereotype is that Asian Americans are “perpetual foreigners” who are not truly “American” (Huynh et al., 2011; Lee et al., 2009, p. 69; Wu, 2002). Research suggests media can influence adoption of this stereotype at the subconscious level (Yogeeswaran et al., 2012). Harboring these implicit beliefs, in turn, may encourage discriminatory acts against Asian Americans, for example in hiring (Yogeeswaran & Dasgupta, 2010).

## Project Implicit

Project Implicit (Nosek et al., 2010) is commonly used to evaluate media effects on racial bias. The tool, available publicly online, measures explicit (i.e., conscious) attitudes via self-report. It also measures implicit (i.e., unconscious) bias via the Implicit Association Test (IAT). The IAT operationalizes implicit bias by comparing reaction times when associating concepts with various social groups (see Supplemental Appendix I). Recently, researchers have utilized Project Implicit data to discern what Payne et al. (2017) describe as the “bias of crowds”—average levels of bias at the regional level, which may capture important aspects of the cultural context (Blair & Brondolo, 2017; Payne et al., 2017). Several studies have identified that the “bias of crowds” is responsive to media representations and social shocks (Inbar et al., 2016; Sawyer & Gampa, 2018; Tukachinsky et al., 2015). For example, Sawyer and Gampa (2018) showed small population-level decreases in anti-Black bias among Whites were associated with key moments in the media’s coverage of the Black Lives Matter movement from 2014 to 2016. Inbar et al. (2016) found slight increases in implicit antigay bias during the Ebola crisis, suggesting public concern over epidemics may affect unconscious social attitudes. Population aggregate levels of racial bias predict inequities in state Medicaid expenditures (Leitner et al., 2018), school discipline (Riddle & Sinclair, 2019), and a variety of adverse health outcomes (Hehman et al., 2018; Leitner et al., 2016a, 2016b; Orchard & Price, 2017). Taken together, evidence suggests that media representations can influence aggregate-level bias, which in turn is associated with important social and health outcomes. However, to our knowledge, the effects of coronavirus media coverage on aggregate bias against Asian Americans are yet unexplored.

## Purpose of the Present Study

Using Project Implicit “Asian IAT” data from 2007–2020, we examined longitudinal trends in bias against Asian Americans relative to European Americans before and during the COVID-19 pandemic. Our study aims were threefold: (1) to describe longitudinal trends in racial bias toward Asian Americans over the 13 years prior to the COVID-19 pandemic; (2) to test for changes in trends on and after March 8, when there was a discernible tone shift in conservative social and news media, characterized by an increase in terms that are stigmatizing to Asian Americans (Rizzuto, 2020); and (3) to explore whether any changes in trends would be differential by political orientation given that conservative individuals may have greater exposure to conservative media (Stroud, 2008). We hypothesized as follows:

**Hypothesis 1:** In the period prior to this media tone shift, bias against Asian Americans decreased over time, which we hypothesized based on documented trends in other forms of bias (Charlesworth & Banaji, 2019);
**Hypothesis 2:** After the March 8 increase in stigmatizing language toward Asian Americans (Rizzuto, 2020), trends in bias over time would flatten or reverse to a positive trend.
**Hypothesis 3:** Given that the tone shift occurred in conservative media outlets, any change in the trends in bias toward Asian Americans would be stronger among those identifying as conservative due to greater exposure to conservative media sources (Stroud, 2008).

## Method

### Data

We downloaded data from *n* = 339,063 non-Asian respondents of the Project Implicit “Asian IAT” from 2007–2020 (Supplemental Appendix I) (Nosek et al., 2010). These data were broken into two data sets. The first data set, spanning the period from January 1, 2007, to February 10, 2020, was designed to provide general information about trends in bias over time prior to our Aim 2 study period (Aim 1). The second data set begins following the February 11, 2020, WHO guidance for media outlets to avoid stigmatizing language when referring to the novel coronavirus and runs through March 31, 2020, the latest date in available Project Implicit data. This second data set was designed to ascertain and formally test whether there was a structural break in the trend in bias prior to, versus after, the March 8 media tone shift (Aim 2), and to ascertain if any such trend shift was differential by political affiliation (Aim 3).

### Study Outcome

*Implicit Americanness Bias* is the comparative ease with which respondents associate “Asian American” versus “European American” faces with American or foreign symbols. Scores range from approximately −2 to 2, with negative values indicating a bias that Asian Americans are more American than European Americans (herein, “pro-Asian bias”), a score of zero indicating no bias, and positive values indicating a bias that European Americans are more American than Asian Americans (herein, “pro-White bias”).

### Covariates

The average characteristics of the individuals who take Project Implicit tests/surveys vary on any given day. Failure to account for these compositional changes can bias estimates. Thus, we constructed the following variables to adjust for compositional effects, which were included in regression models for Aims 2 and 3 unless otherwise specified:

1. *Age*: Respondents’ approximate age at the time of taking the IAT, specified as a continuous variable.
2. *Gender*: Whether a respondent self-identified as a (transgender or cisgender) man, woman, or gender nonbinary (“genderqueer,” “genderfluid,” “nonbinary,” or 2+ genders).
3. *Educational attainment*: Specified as a vector of 14 highest degree attainment categorical indicators to allow a flexible functional form for the effects of education.
4. *U.S. citizen*: Whether or not the participant is a U.S. citizen.
5. *Weekday*: Specified as a vector of dummy variables representing the specific day of the week that a respondent took the IAT.
6. *Political identification*: Self-reported political identification, measured on a 7-point Likert-type scale ranging from a score of 1 (*strongly conservative*) to 7 (*strongly liberal*). We adjusted for political identification in Aim 2, and used as a stratification variable in Aim 3.

We did not adjust for race but rather conducted three subgroup analyses on all non-Asians (i.e., did not self-identify as either East Asian or South Asian), Whites, and non-White non-Asians (i.e., non-Asian minoritized). For detailed information and summary statistics about all study measures, see Supplemental Appendix II.

### Statistical Analyses

#### Aim 1 Analysis

We first described the general trend in bias toward Asian Americans from January 1, 2007, to February 10, 2020. To this end, we visually ascertained overall trends in bias toward Asian Americans among non-Asians by using Stata’s local polynomial function (“lpolyci”) to fit daily 7-day bias averages (StataCorp, 2019). The flexible functional form allowed us to visually inspect both general trends over time and the stability of these trends.

#### Aim 2 Analysis

Second, we sought to determine whether there was a change in the trend of Implicit Americanness Bias on March 8, when there was a large increase in conservative media outlets and elected officials utilizing the terms “Chinese virus” and “Wuhan virus.” We used data from February 11, 2020 (the date the WHO provided guidance regarding how to discuss the novel coronavirus), to March 31, 2020 (the last date for which Project Implicit data are available). This constituted a period exactly 26 days before and 24 days on and after the tone shift on March 8. We chose this tighter time frame to avoid conflating the effect of interest with the effects of other major media events. Consistent with Bernal et al. (2017), to test for a statistically significant change in trend, we regressed Asian American bias on a running variable indicating the date an individual took the test, an indicator variable representing whether that date was in the pre period or post period, and a variable for the statistical interaction between the running variable and indicator variable.^1^ We included controls to account for compositional differences of survey takers over time and repeated the analysis within each of our three racial subgroups. The statistical significance and magnitude of the coefficient on the interaction term indicate whether and to what extent the slope during the pre period is significantly different from the slope during the post period. Our model for discerning a change in trend was the following:

$$\begin{array}{l} Bias_{it}=\alpha +\beta_{1}\left(Day\;of\;Test_{it}\right)+\beta_{2}\left(PostMarch7_{it}\right)+\\ \;\;\beta_{3}\left(Day\;of\;Test\;x\;PostMarch8_{it}\right)+X_{it}+\varepsilon_{it}. \end{array}$$

*Bias* represented the outcome measure for the type of bias being predicted from the four assessed.

- *Day of Test* was a running variable which represented the day of a given Project Implicit test relative to the date of the March 8 media tone shift.^2^
- *PostMarch7* was a dichotomous indicator of whether a test was taken on or after March 8, the day of the media tone shift.
- *Day of Test × PostMarch7* was an interaction term created by taking the product of the running variable and indicator variable described above.
- *X_it_* was a vector of covariate controls (age, gender, educational attainment, U.S. citizen, weekday, and political identification).

This model assumes proper model specification and no unmeasured time-variant confounding. In this model, the coefficient on *Day of Test* represented the estimated time-trend slope during the pre period. We expected this coefficient to be negative. The coefficient on the interaction term was the primary coefficient of interest and can be interpreted as representing the trend change in bias toward Asian Americans on March 8. We expected this interaction coefficient to be greater than zero, indicating a positive trend shift. Thus, we formally tested for its statistical significance against the null hypothesis that the interaction coefficient is zero. Importantly, these interaction coefficients do not provide an indication of where the intercepts fall on either side of the March 8 discontinuity. We thus constructed time-series plots to supplement and further review any of the statistically significant results. We repeated the analysis approach described above for three measures of explicit bias in Supplemental Appendix IV.

#### Aim 3 Analysis

To assess if any change in trend was larger for conservatives (who may have greater exposure to conservative media (Stroud, 2008)), we stratified and repeated our Aim 2 analyses, predicting the trend change in Implicit American Bias within five political ideology subgroups.^3^ We then compared the interaction coefficients from these regressions to see if trend shifts were stronger for certain subpopulations.

## Results

### Aim 1: Trends in Bias Toward Asian Americans From 2007–2020

**Figure 1.**
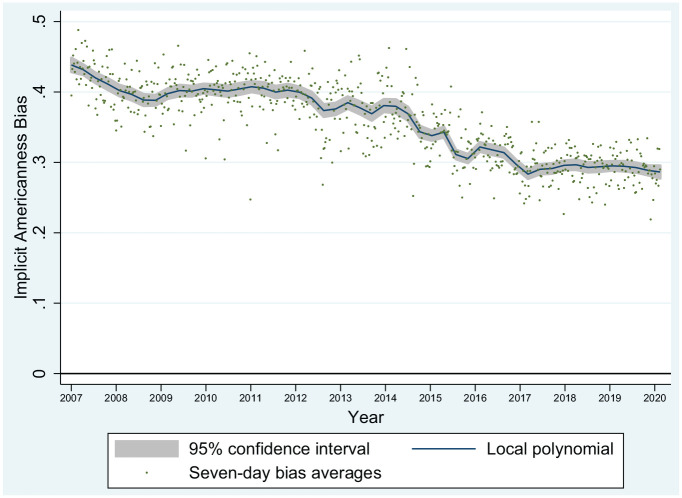
Trends in Implicit Americanness Bias toward Asian Americans, among all non-Asians (*n* = 294,451) from January 1, 2007, to February 10, 2020. *Note.* Sample restricted to respondents who did not self-identify as either East Asian or South Asian. Trends fitted with local polynomial function using a Gaussian bandwidth and Epanechnikov kernel. Horizontal line (zero) indicates neutral bias; below the line indicates pro-Asian bias; and above the line indicates pro-White bias.

From January 1, 2007, to February 10, 2020, there were *n* = 294,451 tests of Implicit Americanness Bias. As depicted in the local polynomial in Figure 1, Implicit Americanness Bias was generally positive (indicating a pro-White bias) but decreased over time from an initial value of 0.44 on January 1, 2007, to a final value of 0.28 on February 10, 2020. Notably, there were brief periods of increase between 2007 and early 2016, but after approximately March 2016, this form of bias appears to have consistently either held steady or declined. Given the relative smoothness of the local polynomial (particularly in the past 4 years), we determined that simple regression was a practicable strategy for delineating overall trends and regressed daily average bias on day of test. We found that over this 13-year time period, Implicit Americanness Bias decreased at a rate of approximately 0.000033 (*SE* = 0.000000573) points per day (95% confidence interval [CI; −0.00034, −0.00032]). This corresponds to approximately 0.001 points per month or 0.012 points per year.

### Aim 2: Trend Changes in Bias Toward Asian Americans on March 8, 2020

From February 11 to March 31, 2020, there were *n* = 4,411 tests of Implicit Americanness Bias. Characteristics of respondents were similar before and after the March 8 media shift (Supplemental Appendix III). As depicted in Table 2, the results indicated a statistically significant, positive trend reversal in Implicit Americanness Bias among all non-Asians (0.0053, 95% CI [0.0020, 0.0087]), and among Whites specifically (0.0042, CI [0.0004, 0.0080]). Results suggested that, in the period from February 11 to March 7, 2020, bias was diminishing at a rate of approximately 0.0037 points per day among non-Asians but began increasing at a rate of 0.0017 points per day on March 8, and increased by approximately 0.041 points between March 8 and March 31. Combining these results from simple regression reported in our Aim 1 analysis, we found that after March 8, Implicit Americanness Bias grew enough to offset more than 3 years of prior declines.^4^

**Table 2.** Adjusted Regression Models Ascertaining Trend Reversal in Implicit Americanness Bias Before and After March 8, 2020, for All Non-Asian, White, and Non-White Non-Asian Respondents.

Group	Coefficient	Implicit Americanness Bias
All non-Asians (*n* = 4,041)	Days from March 8, 2020	−0.00366*** (0.000797)
	Interaction	0.00533** (0.00167)
Whites (*n* = 3,035)	Days from March 8, 2020	−0.00378*** (0.00101)
	Interaction	0.00418* (0.00189)
Non-White non-Asians (*n* = 1,006)	Days from March 8, 2020	−0.000748 (0.00260)
	Interaction	0.00585 (0.00341)

*Note.* Sample restricted to respondents who did not self-identify as either East Asian or South Asian. The second and third samples were further restricted to Whites and non-Whites, respectively. All regression models adjusted for age, gender, educational attainment, U.S. citizenship, weekday of test, and political identification. Robust standard errors shown in parentheses.

* *p* < .05. ^**^p < .01. ^***^p < .001.

As noted above, we reviewed all statistically significant regression results with time-series plots to visually ascertain if pretrends and posttrends aligned with hypothesized expectations. Specifically, given our hypothesis of trend reversals, we expected these plots to evidence three features:

1. A negative slope prior to March 8.
2. A jump in the intercept on March 8 such that the line on the left concludes at a lower point than the line on the right begins.
3. A positive slope after March 8.

**Figure 2.**
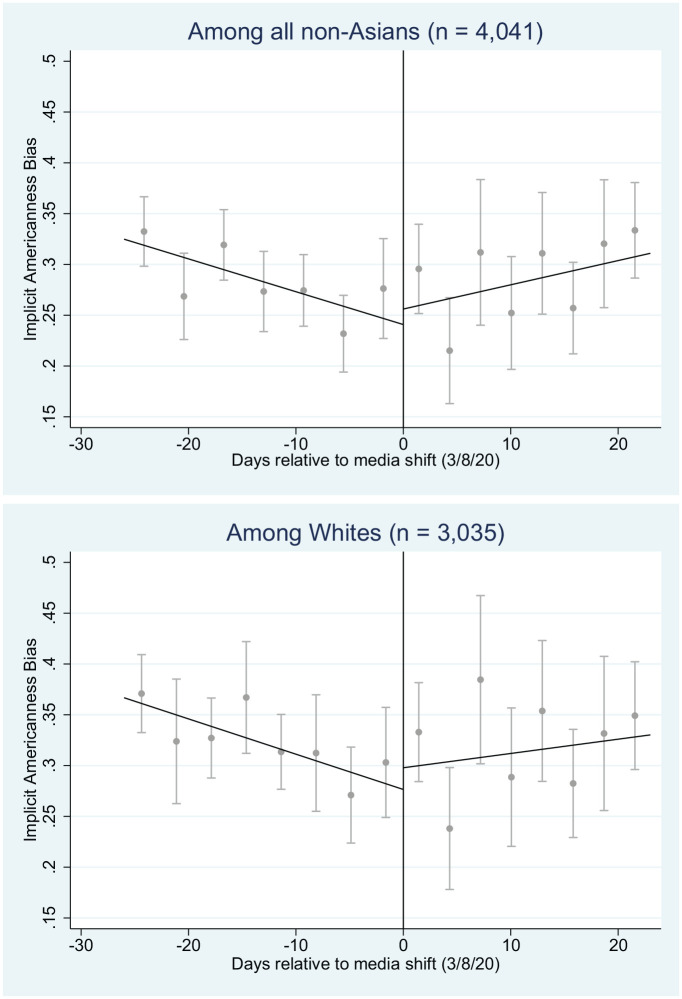
Trends in Implicit Americanness Bias before and after March 8, 2020. *Note.* Sample restricted to respondents who did not self-identify as either East Asian or South Asian. Dots show average bias over 3 days. Bands show 90% confidence intervals.

Reviewing the time-series plots (Figure 2), we saw our hypothesized pattern among all non-Asians (top panel) and Whites (bottom panel). In both cases, before March 8, 2020, the slope was negative; at March 8, we saw a jump in bias; and after March 8, we saw a positive slope. This suggested that when media entities began using stigmatizing terms like “Chinese virus,” Implicit Americanness Bias began to increase.

### Aim 3: Differential Strength of Trend Reversal Effects by Political Ideology

**Figure 3.**
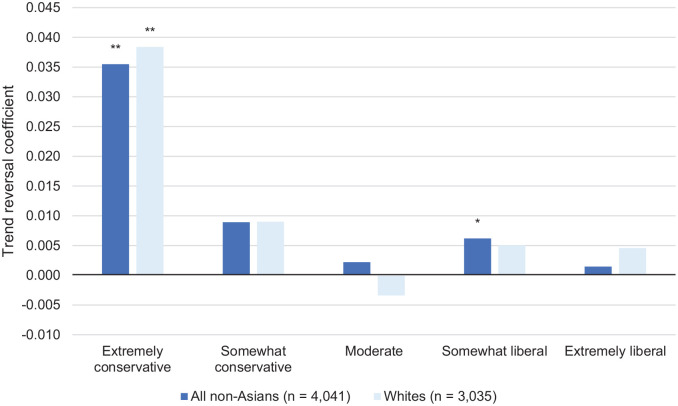
Size and statistical significance of trend reversal coefficients by political identification. *Note.* Trend reversal coefficient represents the interaction of Day of Test and our indicator for whether or not the test was taken after March 7. Sample restricted to respondents who did not self-identify either East Asian or South Asian. Horizontal line indicates neutral bias; below the line indicates pro-Asian bias; and above the line indicates pro-White bias. All regression models adjusted for age, gender, educational attainment, U.S. citizenship, and weekday. ^*^p < .05. ^**^p < .01. ^***^p < .001.

We next determined whether the trend reversal coefficient was more pronounced for individuals who described themselves as conservative, as compared to individuals of other political identifications. This inquiry was rooted in the assumption that conservatives were more likely to consume conservative media (Stroud, 2008) where stigmatizing terminology increased (Rizzuto, 2020). As depicted in Figure 3 below, trend reversals were much larger among extreme conservatives than among members of any other political subgroup.

## Discussion

The objectives of this study were to estimate long-term trends in bias toward Asian Americans from January 1, 2007, to March 31, 2020, and to empirically test whether trends in bias shifted on March 8, 2020, following a large increase in the use of stigmatizing terms like “Chinese virus” by conservative media outlets (Rizzuto, 2020). We found that, among non-Asians, Implicit Americanness Bias—or the belief that Asian Americans are more foreign and less American compared to European Americans—fell steadily from 2007 to early 2020. However, in our models, Implicit Americanness Bias began to increase for all non-Asians, and for Whites specifically, on March 8, with stronger trend reversals observed among individuals who identified as more conservative. To put these results in perspective, we estimate that in the approximately 3-week period from March 8 to March 31, not only did aggregate-levels of Implicit Americanness Bias among non-Asians grow after 13 years of fairly steady decline, it also grew enough to offset more than 3 years of prior declines. Importantly, it has been suggested that even small increases in aggregate implicit bias can have large societal implications because they represent simultaneous impacts on many people and/or repeated impacts on individual people (Greenwald et al., 2015).

This study makes three critical contributions to extant literature on media effects and racial bias. First, it adds to the burgeoning literature on sources of bias and provides, to our knowledge, the first empirical assessment of determinants of aggregate bias toward Asian Americans. Second, it further demonstrates the utility of interrupted time-series methods for estimating trend changes in bias. Finally, this research sounds an alarm about the potential impact of stigmatizing language in news and social media. After March 8, myriad organizations criticized conservative media entities for using “Chinese virus” and related terminology, warning that this stigmatizing language could increase bias toward Asian Americans (Human Rights Watch, 2020; Tavernise & Oppel, 2020). This evidence suggests it did.

### Methodological Considerations and Study Limitations

As we consider the potential impact of stigmatizing media on racial bias, it is important to note that in this study, we focus on aggregate-level bias, which may capture an emergent construct distinct from its individual-level analog (Blair & Brondolo, 2017; Hehman et al., 2019; Payne et al., 2017). Our finding, then, is not that a media tone shift increased any given individual’s biases. Instead, we find evidence that when conservative media increased their use of stigmatizing terminology, it increased *collective* bias, especially for conservatives. Critically, our measure of implicit bias captures *not* generalized *anti-Asian bias*, but rather the perception that Asian Americans are *less American*, consistent with the “perpetual foreigner” stereotype (Lee et al., 2009) and media framing of the coronavirus as a “Chinese” (i.e., *not American)* virus.

This evidence should be examined in light of several limitations. First, Project Implicit respondents are self-selected, limiting the external validity of our estimates. Relatedly, while we adjusted for key demographics in our statistical models, and while we find evidence that average characteristics of test takers are very similar in our preperiod and postperiod (see Supplemental Appendix III), we cannot rule out the possibility that our results are influenced by *unmeasured* compositional changes in test takers over time.

Another key consideration is that to estimate the causal effect of the media shift, we must assume that no other social phenomena shifted trends in Implicit Americanness Bias on or around March 8. One alternative explanation for these findings comes from “behavioral immune system” theory, which posits that fears and biases may be generally heightened during a pandemic, resulting in increased prejudice against stigmatized groups due to historical blaming of these groups for the spread of disease (Schaller & Park, 2011). There is, to our knowledge, no research that suggests that such responses will differ by political ideology. However, we find that individuals who identified as conservative evidenced larger trend reversals than others—a result strongly predicted by media effects theory (Wetts & Willer, 2019). This suggests that increases in stigmatizing language in conservative media on March 8, rather than generalized bias toward stigmatized groups during the pandemic, was a primary driver of the trend reversal in Implicit Americanness Bias. Future work could consider whether media effects and behavioral immune system responses interact such that media effects are more pronounced in times of pandemic threat.

### Implications for Health and Well-Being of Asian Americans

Rhetoric is not harmless. This analysis suggests that the use of terms like “Chinese virus” had an immediate, measurable impact on our collective biases. Specifically, this stigmatizing terminology more deeply entrenched the notion that Asians are “perpetual foreigners”—a pernicious stereotype with troubling historical roots and measurable real-world consequences (Huynh et al., 2011; Lee et al., 2009; Wu, 2002). Mounting evidence suggests that area-level rates of bias toward another stigmatized group, Black Americans, may contribute to health inequities (Hehman et al., 2018; Leitner et al., 2016a, 2016b; Leitner et al., 2018; Orchard & Price, 2017; Riddle & Sinclair, 2019). To our knowledge, this is the first study to examine bias toward Asian Americans in the aggregate, the health effects of which warrant close examination. Research demonstrates that the implicit belief that Asian Americans are foreign predicts hiring discrimination (Yogeeswaran & Dasgupta, 2010), and that economic disadvantage can contribute to poor health outcomes for Asian Americans (De Castro et al., 2010). Moreover, Asian Americans report chronic day-to-day reminders of the “perpetual foreigner” stereotype (e.g., “Where are you from?” “You speak such good English”; Wu, 2002), and studies suggest that everyday discrimination is associated with poorer mental and physical health outcomes among Asian Americans (Gee et al., 2007; Gee et al., 2009; Huynh et al., 2011). Finally, the increase in violent hate crimes directed at Asian Americans during this time cannot be ignored (Chinese for Affirmative Action and Asian Pacific Policy & Planning Council, 2020). Whether media-driven shifts in aggregate-level Implicit Americanness Bias encourage institutional and individual racism against Asian Americans—and the consequential social, health, and economic impacts—emerge as pressing questions for future research.

### Implications for Policy and Practice

This research would not have been possible without the work of the Atlantic Council’s DFL (Rizzuto, 2020). We hope, therefore, that these findings encourage more funding and public support for media accountability efforts. Armed with knowledge that at least one form of implicit bias is creeping up, we hope researchers will seek interventions that can drive biases down and, eventually, eliminate them from our collective consciousness. Finally, it is critical to note that much of the media featuring stigmatizing terms was generated directly by prominent elected officials, including the president of the United States. Particularly given the evidence presented in this article, we urge elected leaders to avoid language that can inflame biases and thus may threaten the health and well-being of Asian Americans.

## Supplemental Material

HEB_Online_Supp_08.27.20 – Supplemental material for After “The China Virus” Went Viral: Racially Charged Coronavirus Coverage and Trends in Bias Against Asian Americans Click here for additional data file. Supplemental material, HEB_Online_Supp_08.27.20 for After “The China Virus” Went Viral: Racially Charged Coronavirus Coverage and Trends in Bias Against Asian Americans by Sean Darling-Hammond, Eli K. Michaels, Amani M. Allen, David H. Chae, Marilyn D. Thomas, Thu Nguyen, Mahasin M. Mujahid and Rucker C. Johnson in Health Education & Behavior

## Notes

1. While it was feasible to add higher order terms in interrupted time-series models, we found that such models largely picked up noise inherent in Project Implicit’s data. Moreover, as depicted in Figure 1, we saw evidence that, generally, Implicit Americanness Bias followed a linear trend over time. We thus opted for linear models.

2. So, for example, a test on March 7 would be coded as “−1,” a test on March 8 would be coded as “0,” a test on March 9 would be coded as “1,” and so on. Values range from −26 to 23, representing a 50-day time period from February 11 to March 31, 2020.

3. The five political ideology subgroups were constructed using Project Implicit’s measure of political ideology—a Likert-type measure ranging from 1 (*conservative*) to 7 (*liberal*). Individuals were assigned to the five groups accordingly: extreme conservatives (those who gave a response of 1 on Project Implicit’s 7-point Likert-type measure); somewhat conservatives (responses of 2 or 3); moderates (4); somewhat liberals (5 or 6), and extreme liberals (7).

4. This comparison relied on the estimated value of the interaction coefficient (0.0053). Using the 95% CI around the coefficient, we estimated that even at the lower bound of the confidence interval (0.0020), we would expect that the trend shift more than halved the rate of daily bias decline experienced in the period from February 11 to March 7 (−0.0037) and would more than reverse the typical rate of daily bias decline experienced from January 1, 2007, to February 10, 2020 (−0.000033).
