# Supplementary material for: After “The China Virus” Went Viral: Racially Charged Coronavirus Coverage and Trends in Bias Against Asian Americans
Source: Health Educ Behav. 2020 Sep 10;47(6):870–9. doi: 10.1177/1090198120957949 (PMC7488172; doi:10.1177/1090198120957949)
Supplement: HEB_Online_Supp_08.27.20 – Supplemental material for After “The China Virus” Went Viral: Racially Charged Coronavirus Coverage and Trends in Bias Against Asian Americans [file HEB_Online_Supp_08.27.20.pdf]

SUPPLEMENTAL MATERIAL

After "the China Virus" went viral: Racially charged Coronavirus coverage and trends in bias against Asian Americans

Appendix I: The Implicit Association Test (IAT)

IATs are publicly available at https://implicit.harvard.edu/implicit/selectatest.html

Myriad tests exist, and each utilizes keyboard tasks to measure the ease and accuracy with which respondents link groups with specific concepts as a proxy for bias. To take the Asian IAT, respondents visited the link above and select the "Asian IAT" button. Upon doing so, participants were greeted with the following explanatory screen showing the six foreign landmarks, American landmarks, "Asian American" faces, and "European American" faces that were used to ascertain their implicit associations. They then completed two training task (Parts 1 and 2) which helped them familiarize themselves with the keyboard by asking them to press E or I if they see Asian- or European American faces (Part 1) or American or foreign landmarks (Part 2).

Sample Asian IAT test screenshots: Introductory explanation and training tasks (parts 1 and 2)

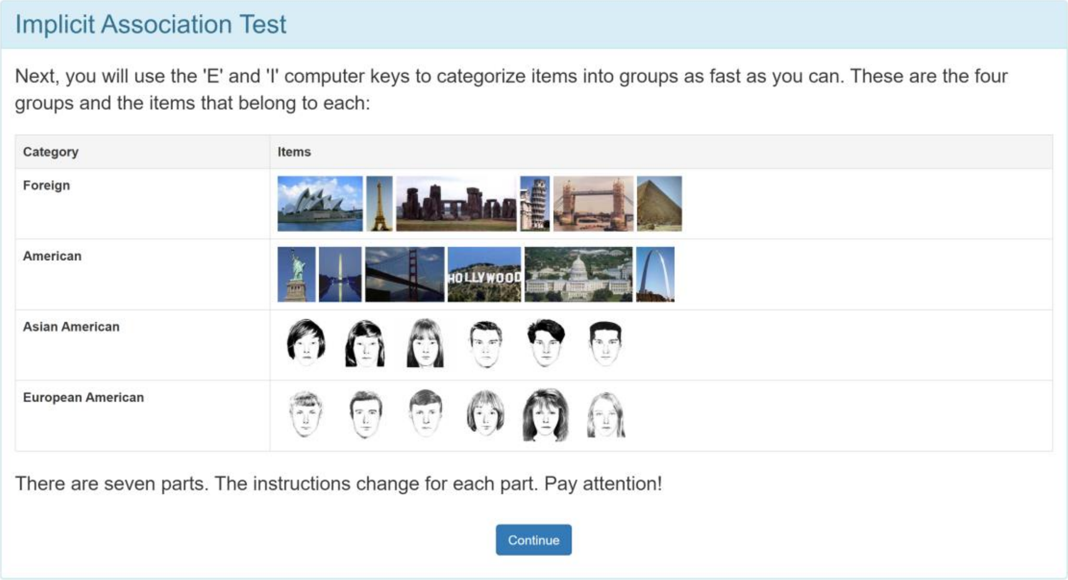

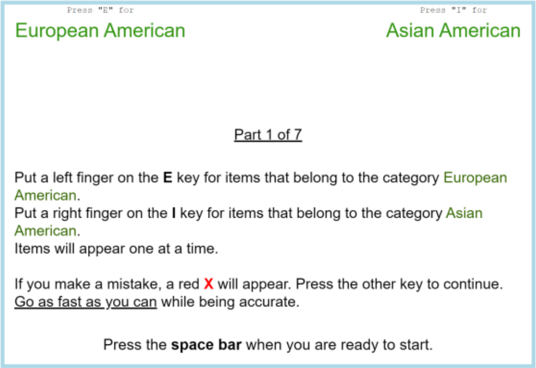

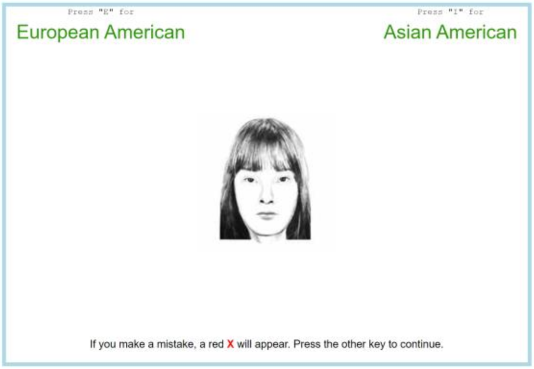

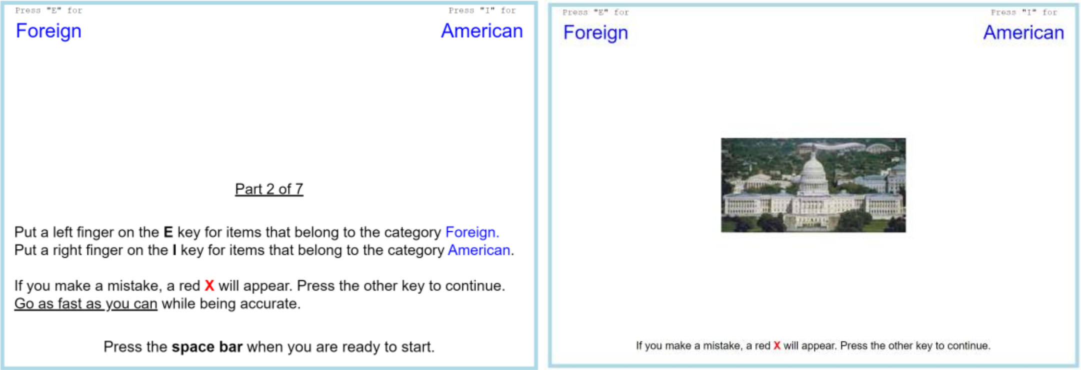

Next, they completed two tasks (Parts 3 and 4) where they were asked to press one key if they saw Foreign landmarks *or* European American faces, and to press a different key if they saw American landmarks *or* Asian American faces. This was meant to ascertain the ease with which they associated European Americans with foreignness, and Asian Americans with Americanness.

Sample Asian IAT test screenshots: Part 3 (identical to part 4)

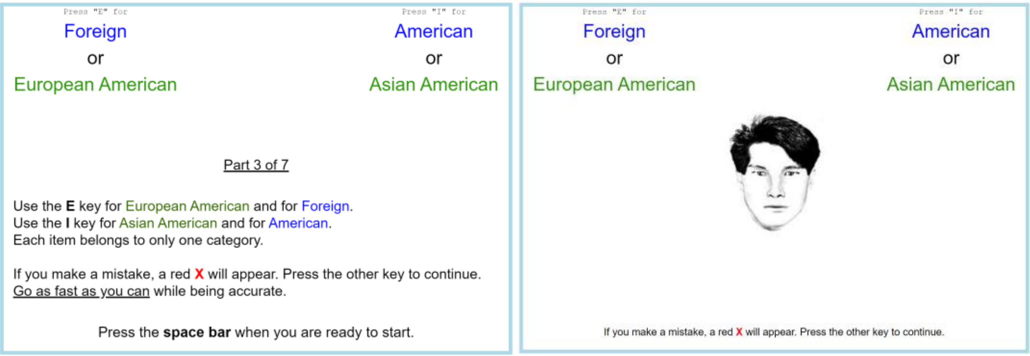

Participants then completed a training task (Part 5) designed to help them reorient to new keyboard letter meanings: E was now associated with Asian American faces and I with European American faces.

Sample Asian IAT test Screenshots: Part 5

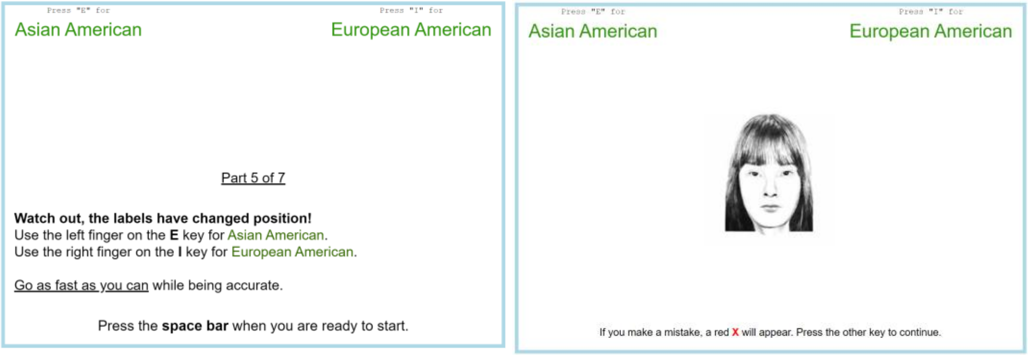

Finally, participants completed two tasks (Parts 6 and 7) in which they were instructed to press E if they saw foreign landmarks *or* Asian American faces, and press I if they aw American landmarks *or* European American faces.

Sample Asian IAT Test Screenshots: Part 6 (identical to Part 7)

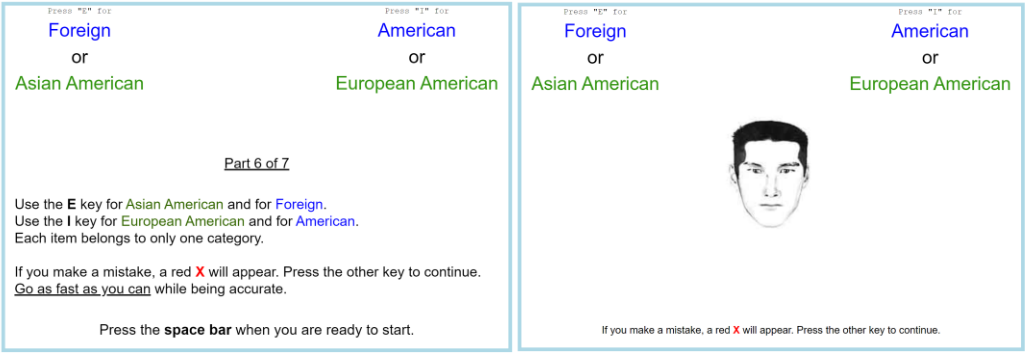

Participants were scored on how easily (accurately and quickly) they completed Parts 3 and 4 (on average) versus parts 5 and 6 (on average). For more detail on how IAT scores are created, and on the IAT more generally (including the randomization of tasks and keyboard meanings), see Greenwald, Nosek, and Banaji (2003).

Appendix II: Detailed information about study measures

Outcome Measures:

Implicit Americanness Bias: As detailed in Appendix I, respondents were tasked with making associations between White vs. Asian faces with American (e.g., bald eagle, statue of liberty) vs. foreign (e.g., iconic buildings in countries around the world) symbols. Faster and more accurate associations of "European American" faces with American symbols and/or faster associations of "Asian American" faces with foreign symbols was indicative of an American-is-White/Asian-is-foreign implicit bias. Negative scores indicated a bias that Asian Americans are more American than European Americans, a score of zero indicates no implicit bias, and positive scores indicated a bias that European Americans are more American than Asian Americans.

As reported in Appendix IV, we repeated our analysis on Implicit Americanness Bias on three measures of explicit bias which are discussed below.

Explicit Americanness Bias: To construct this bias measure, we took the difference between two attitudinal measures. In the first, respondents were asked to indicate, on a seven-point Likert scale, "In your mind, how American are Asian Americans?" A score of 1 indicated feeling Asian Americans were "Strongly foreign" in a given respondents' mind, and a 7 indicated feeling Asian Americans were "Strongly American." In the second measure, responds were asked the same question about "White Americans." By differencing the scores, we ascertained the extent to which each respondent explicitly felt White Americans were "more American" than Asian Americans. Scores ranged from -6 to 6. Negative scores indicated a bias that Asian Americans were more American than White Americans, a score of zero indicated no implicit

bias, and positive scores indicated a bias that White Americans were more American than Asian Americans. Note, this is the only one of the four measures that referred to “White Americans” as opposed to “European Americans,” potentially limiting comparability with the other measures.

Explicit Warmth Bias: In much the same manner that the IAT ascertained sentiments about groups’ Americanness, it also ascertained respondents’ feelings of warmth toward Asian Americans and European Americans, asking “How warm or cold do you feel toward Asian Americans” and, in a separate question “toward European Americans?” Scores ranged from 0 (“extremely cold”) to 10 (“extremely warm”). Following a common research approach employed on these measures we differenced the scores to discern the extent to which each respondent felt warmer toward European Americans than Asian Americans (ranging from -10 to 10) (Hehman et al., 2019; Hehman et al., 2017; Leitner et al., 2016a, 2016b; Leitner et al., 2018). Negative scores indicated greater feelings of warmth toward Asian Americans as compared to European Americans, a score of zero indicated no bias, and positive scores indicated greater feelings of warmth toward European Americans as compared to Asian Americans.

Explicit Preference Bias. Respondents were asked to report their preference for Asian Americans or European Americans on a seven-point Likert scale where a 1 indicated “I strongly prefer Asian Americans” and a 7 represented strongly preferring “European Americans.” We subtracted 4 from this measure such that, consistent with the measures above, negative scores indicated preference for Asian Americans as compared to European Americans, a score of zero indicated no bias, and positive scores indicated greater preference for European Americans as compared to Asian Americans.

Covariates:

The average characteristics of the individuals who take Project Implicit tests can vary on any given day. Failure to adjust for these "compositional effects" can bias estimates. Thus, in order to adjust for compositional effects, via subgroup analyses and regression controls, we constructed the following variables:

(1) Race/ethnicity: As noted above, we restricted all of our analyses to non-Asian individuals based on self-reported race (dropping individuals who indicate being entirely or partially East-Asian or South-Asian). In certain sub analyses, we further restricted our analysis samples to White individuals, and in certain others, we restricted to only non-White individuals. In essence, this amounted to reviewing bias trends for non-Asian Whites and for non-White non-Asians.

(2) *Age*: We only utilized age information to analyze January 2020 – March 2020 data (Aims 2 and 3). Thus, we discerned age by subtracting an individual's birth year from 2020. Because our analysis covered a small, three-month window, we believed more precise methods for estimating age were unnecessary.

(3) *Gender*: We constructed a three-factor gender variable (woman, man, nonbinary) by combining information from two separate measures: a self-selected gender identity variable and a birthsex measure. Individuals were coded according to their gender identity. For example, "woman" included cisgender women and transgender women. Those who indicated "gender fluid," "gender queer," "nonbinary," or who indicated more than one gender were assigned "nonbinary." In the rare instances where gender identity information was missing, birthsex was substituted.

(4) Educational attainment: Educational attainment was self-reported from among 14 categories:

1. elementary school
2. junior high
3. some high school
4. high school graduate
5. some college
6. associate's degree
7. bachelor's degree
8. some graduate school
9. master's degree
10. J.D.
11. M.D.
12. Ph.D.
13. other advanced degree
14. M.B.A.

(5) *U.S. Citizen*: Respondents selected their country of citizenship from a dropdown. This information was recoded to create a measure indicating whether or not a given participants was a U.S. Citizen. While citizenship is only one aspect of an individual's geographic identity, we believed it was a salient aspect when ascertaining how "American" an individual perceives members of various racial and ethnic groups to be.

(6) *Political identification*: Respondents self-reported their political identification on a 7-point Likert scale ranging from a score of 1 (“strongly conservative”) to 7 (“strongly liberal”).

Weekday: Project implicit shared the weekday during which a given test was taken, which we included as a vector of dummy variables for which day of the week the test was taken. We adjusted for this information in our models to address at least one source of cyclical variation in who takes Project Implicit tests.

Appendix III: Descriptive findings

Characteristics for the two study samples for Aim 2 and Aim 3 were relatively similar. For example, the average age of Project Implicit respondents in the first sample (January 2007 – February 10, 2020) was 28.8 years old, and the average age in the second sample (February 11 – March 31, 2020) was 29.9 years old. The table below summarizes other sample characteristics, including average values on the four bias measures for non-Asian individuals in both time periods.

Table A1. *Summary statistics for Aim 1 (Jan. 1, 2007 – Feb. 10, 2020) and Aim 2 (Feb. 11, 2020 – Mar. 31, 2020) (n=339063)*

	Aim 1		*Aim 2*	
Variable	N	Mean	N	Mean
Age	326,038	27.77	4,690	29.93
White	334,205	71%	4,858	74%
Black	334,205	6%	4,858	9%
Hispanic*	321,468	11%	4,674	15%
US Citizen	326,374	84%	4,773	85%
Female	332,063	61%	4,848	64%
Education level:				
Elementary school	324,091	0%	4,648	0%
Junior high	324,091	2%	4,648	2%
Some high school	324,091	13%	4,648	9%
High school graduate	324,091	9%	4,648	10%
Some college	324,091	29%	4,648	24%
Associate’s degree	324,091	7%	4,648	8%
Bachelor’s degree	324,091	17%	4,648	19%
Some graduate school	324,091	7%	4,648	7%
Master’s degree	324,091	9%	4,648	12%
J.D.	324,091	2%	4,648	1%
M.D.	324,091	1%	4,648	1%
Ph.D.	324,091	3%	4,648	3%
Other advanced degree	324,091	1%	4,648	1%
M.B.A.	324,091	1%	4,648	1%
Bias Measures:				
Implicit Americanness Bias	294,451	0.35	4,411	0.28
Explicit Americanness Bias	294,156	0.48	4,513	0.34
Explicit Preference Bias	298,491	0.12	4,522	0.02
Explicit Warmth Bias	108,469	-0.12	4,551	-0.16

Note: Sample restricted to Project Implicit respondents who did not self-identify as either South-Asian or East-Asian.

* includes respondents of any race who identify as Hispanic

Even relative to their ranges, Implicit Americanness Bias (ranging from -2 to 2) and Explicit Americanness Bias (ranging from -3 to 3) were markedly higher, in both study periods, than either Explicit Preference Bias (ranging -3 to 3) or Explicit Warmth Bias (ranging -10 to 10). Average levels of bias were generally lower in the latter time period, consistent with the notion that bias generally diminishes over time. The one exception to this trend was Explicit Warmth Bias. While scores on this measure did go down, they trended away from neutrality (zero).

Zooming in on the *Aim 2* data, we also reviewed average characteristics for test takers in the pre-period and post-period to ascertain the potential for aggregate compositional effects. Importantly, we also controlled for each of these potential sources of confounding. As depicted in the table below, average characteristics in the pre -and post- periods were very similar. So, too, were average bias levels.

Table A2. *Summary statistics for Aim 2 Pre-Period (Feb. 11, 2020 – Mar. 7, 2020) and Post-Period (Mar. 8, 2020 - Mar. 31, 2020) (n=4858)*

	Pre-Period		*Post-Period*	
Variable	N	Mean	N	Mean
Age	2,876	29.84	1,814	30.09
White	2,979	74%	1,879	73%
Black	2,979	9%	1,879	9%
Hispanic*	2,852	15%	1,822	17%
US Citizen	2,928	85%	1,845	85%
Female	2,974	65%	1,874	64%
Education level:				
Elementary school	2,847	1%	1,801	0%
Junior high	2,847	3%	1,801	2%
Some high school	2,847	10%	1,801	8%
High school graduate	2,847	10%	1,801	10%
Some college	2,847	24%	1,801	25%
Associate’s degree	2,847	8%	1,801	8%
Bachelor’s degree	2,847	19%	1,801	21%
Some graduate school	2,847	6%	1,801	8%
Master’s degree	2,847	13%	1,801	12%
J.D.	2,847	1%	1,801	2%
M.D.	2,847	1%	1,801	1%
Ph.D.	2,847	3%	1,801	2%
Other advanced degree	2,847	1%	1,801	1%
M.B.A.	2,847	1%	1,801	1%
Bias Measures:				
Implicit Americanness Bias	2,690	0.29	1,721	0.28
Explicit Americanness Bias	2,762	0.36	1,751	0.32
Explicit Preference Bias	2,762	0.01	1,760	0.03
Explicit Warmth Bias	2,786	-0.17	1,765	-0.15

Appendix IV: Analysis of trends and trend-changes in explicit bias measures

Here, we reported results from local polynomial charts, regression models, and trend charts estimating trends and trend-shifts in explicit bias measures.

As depicted in Figure A1 below, from January 1, 2007 – February 10, 2020, trends in all three forms of explicit bias were negative overall, but erratic, with many sporadic periods of decrease and increase over time. Explicit Americanness Bias was generally positive, indicating pro-White bias. While Explicit Preference Bias was initially decidedly positive, from 2016 onward, Explicit Preference Bias hovered at around zero, indicating no clear directional bias. Explicit Warmth Bias was generally negative, indicating pro-Asian bias.

Figure A1. *Trends in three measures of explicit bias toward Asian Americans, among all non-Asians (n=307,454), from January 1, 2007, to February 10, 2020*

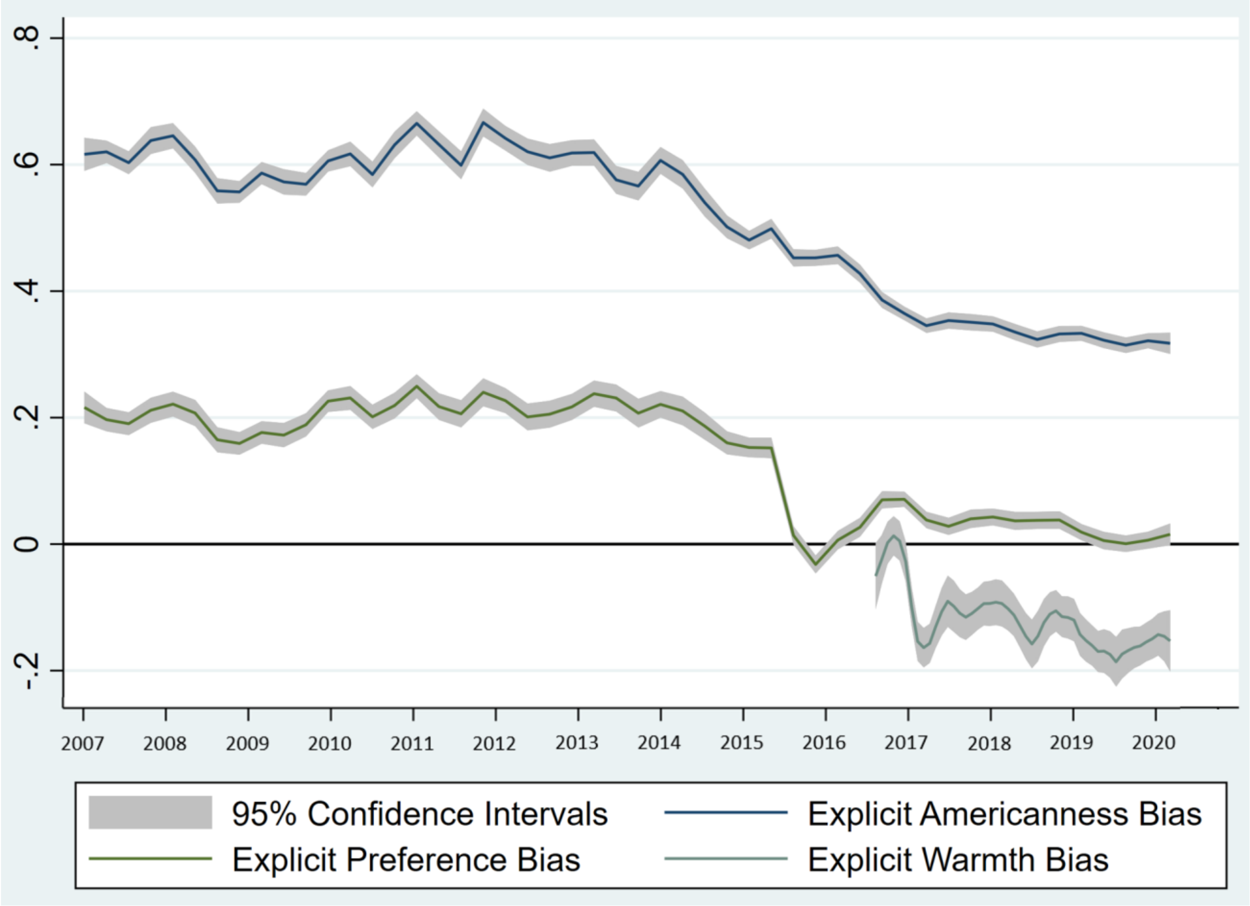

Note: sample restricted to respondents who did not self-identify as either East-Asian or South-Asian.

Trends fitted with local polynomial function using a Gaussian bandwidth and Epanechnikov kernel.

Horizontal line (zero) indicates neutral bias; below the line indicates pro-Asian bias and above the line indicates pro-White bias.

As depicted in Table A3 below, we saw evidence of trend-reversals related to Explicit Americanness Bias among all non-Asians, and related to all three explicit bias measures among non-White, non-Asians.

Table A3. *Adjusted+ regression models ascertaining trend-shifts in three measures of explicit bias before and after March 8, 2020, for all non-Asian, White, and non-White non-Asian test takers*

**Group**		Explicit Americanness	Explicit Warmth	Explicit Preference
**All non-Asians**	Days from 3/8/20	-0.00154	-0.00239	-0.00188
		(0.00289)	(0.00550)	(0.00278)
	**Interaction**	**0.0124***	**0.0135**	**0.00706**
		**(0.00505)**	**(0.00819)**	**(0.00365)**
	n	4170	4192	4169
**Whites**	Days from 3/8/20	-0.00157	0.00237	-0.000831
		(0.00328)	(0.00539)	(0.00332)
	**Interaction**	**0.00745**	**-0.000385**	**0.00232**
		**(0.00599)**	**(0.00823)**	**(0.00409)**
	n	3114	3126	3112
**Non-White non-Asians**	Days from 3/8/20	0.000414	-0.00484	0.000610
		(0.00580)	(0.00802)	(0.00469)
	**Interaction**	**0.0280****	**0.0391****	**0.0151***
		**(0.00858)**	**(0.0127)**	**(0.00721)**
	n	1056	1066	1057

+ All regression models are adjusted for age, gender, educational attainment, U.S. citizenship, weekday of test, and political identification. Robust standard errors shown in parentheses.

Note: all samples restricted to respondents who did not self-identify as either East-Asian or South-Asian. The second and third samples are further restricted to Whites and non-Whites, respectively. Sample sizes fluctuate because not all respondents completed assessments of all bias types.

$*$ p < .05, $**$ p < .01, $***$ p < .001

As with our analysis of implicit bias trend shifts, we further reviewed statistically significant results with trend charts. And as before, we reviewed these trend charts for negative

slopes prior to March 8, a positive jump at March 8 such that the intercept from the left is lower than the intercept from the right, and a positive slope after March 8. As depicted in Figure A2 below, while, in all four cases, the first and third condition are satisfied, the second condition is not.

Figure A2. *Trends in explicit bias measures before and after March 8, 2020*

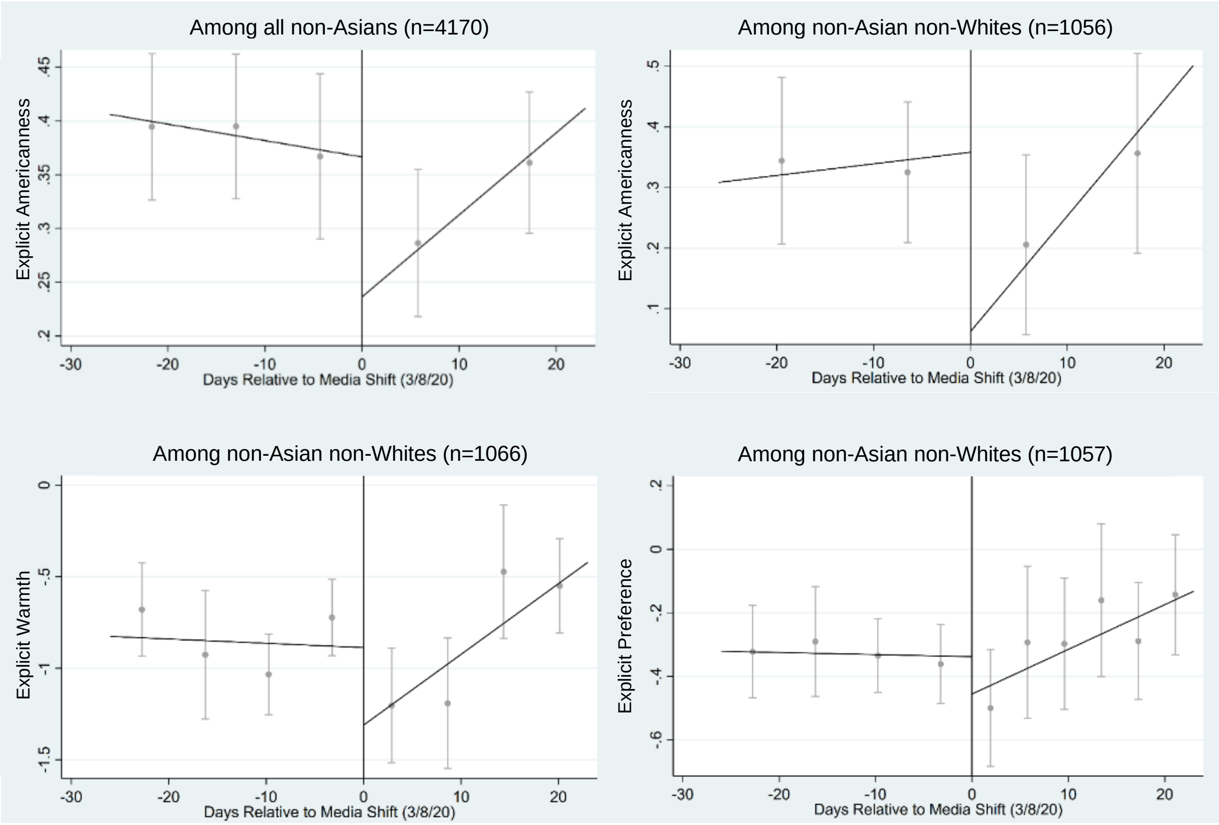

Reviewing these results in total, while there was evidence that Explicit Americanness Bias and Explicit Preference Bias decreased between January 1, 2007, and February 10, 2020, there did not appear to be sufficient evidence to conclude there was a trend reversal in explicit bias on March 8, 2020, for any measure of explicit bias. This stood in contrast to analyses of Implicit Americanness Bias trends which demonstrated more stable decreases over time in the thirteen-year period and showed clear trend reversal effects on March 8. These results may suggest that media effects may more readily impact implicit biases than explicit biases. This may provide insight regarding how best to combat the biasing effects of stigmatizing media.

Particularly given the potential negative impacts of these biases, we believe future research on this topic is warranted.

References

Hehman, E., Calanchini, J., Flake, J. K., & Leitner, J. B. (2019). Establishing construct validity evidence for regional measures of explicit and implicit racial bias. *Journal of Experimental Psychology: General, 148*(6), 1022.

Hehman, E., Flake, J. K., & Calanchini, J. (2017). Disproportionate use of lethal force in policing is associated with regional racial biases of residents. *Social Psychological and Personality Science*, 1948550617711229.

Leitner, J. B., Hehman, E., Ayduk, O., & Mendoza-Denton, R. (2016a). Blacks' death rate due to circulatory diseases is positively related to whites' explicit racial bias: A nationwide investigation using project implicit. *Psychological Science, 27*(10), 1299-1311.

Leitner, J. B., Hehman, E., Ayduk, O., & Mendoza-Denton, R. (2016b). Racial bias is associated with ingroup death rate for Blacks and Whites: Insights from Project Implicit. *Social Science & Medicine, 170*, 220-227.

Leitner, J. B., Hehman, E., & Snowden, L. R. (2018, Jul 2018
2020-03-25). States higher in racial bias spend less on disabled medicaid enrollees. *Social Science & Medicine, 208*, 150.
